# Resectable adenocarcinomas in the pancreatic head: the retroperitoneal resection margin is an independent prognostic factor

**DOI:** 10.1186/1471-2407-8-5

**Published:** 2008-01-14

**Authors:** Arne Westgaard, Svetlana Tafjord, Inger N Farstad, Milada Cvancarova, Tor J Eide, Oystein Mathisen, Ole Petter F Clausen, Ivar P Gladhaug

**Affiliations:** 1 Faculty of Medicine, University of Oslo, Rikshospitalet University Hospital, 0027 Oslo, Norway; 2 Department of Surgery, Rikshospitalet University Hospital, 0027 Oslo, Norway; 3 Pathology Clinic, Rikshospitalet University Hospital, 0027 Oslo, Norway; 4 Biostatistics, Rikshospitalet University Hospital, 0027 Oslo, Norway

## Abstract

**Background:**

The retroperitoneal margin is frequently microscopically tumour positive in non-curative periampullary adenocarcinoma resections. This margin should be evaluated by serial perpendicular sectioning. The aim of the study was to determine whether retroperitoneal margin involvement independently predicts survival after pancreaticoduodenectomy within a framework of standardized assessment of the resected specimens.

**Methods:**

114 consecutive macroscopically margin-free periampullary adenocarcinomas were examined according to a prospective standardized protocol for histopathologic evaluation. The retroperitoneal margin was assessed by serial perpendicular sectioning. The periampullary cancer origin (pancreas, ampulla, distal bile duct or duodenum) was registered prospectively and reevaluated retrospectively. Associations between histopathologic factors were evaluated by Chi-square test, Fisher's exact test, Kruskal-Wallis test, and Mann-Whitney test, as appropriate. Survival curves were calculated by the Kaplan-Meier method and compared using the log-rank test. Associations between histopathologic factors and survival were also evaluated by unadjusted and adjusted Cox regression analysis, including stepwise variable selection, in order to identify factors that independently predict a poor prognosis after periampullary adenocarcinoma resections.

**Results:**

Microscopic resection margin involvement (R1 resection) was present in 40 tumours, of which 32 involved the retroperitoneal margin. Involvement of the retroperitoneal margin independently predicted a poor prognosis (p = 0.010; HR 1.89; CI 1.16–3.08) after presumed curative (R0 and R1) resection. In microscopically curative (R0) resections (n = 74), pancreatic tumour origin was the only factor that independently predicted a poor prognosis (p < 0.001; HR 4.71 for pancreatic versus ampullary; CI 2.13–10.4).

**Conclusion:**

Serial perpendicular sectioning of the retroperitoneal resection margin demonstrates that tumour involvement of this margin independently predicts survival after pancreaticoduodenectomy for adenocarcinoma. Periampullary tumour origin is the only histopathologic factor that independently predicts survival in microscopically curative (R0) resections.

## Background

Resectable primary adenocarcinomas located in the pancreatic head may derive from the pancreatic tissue, the hepatopancreatic ampulla, the distal bile duct or the duodenum, and collectively these cancers may be referred to as periampullary adenocarcinomas [1]. The precise tumour origin is often impossible to determine prior to surgery, and pancreaticoduodenectomy is thus performed for all four types irrespective of tumour origin. Complete tumour removal is one of the most important factors influencing long-term survival after resection [2-6]. However, even after margin-free resection (R0 resection) the recurrence rate is high and the majority of patients succumb to the disease within 5 years [2-6].

The reported proportion of patients having tumour involved resection margins (R1 resection) after pancreaticoduodenectomy varies considerably, in the range 31–85% for pancreatic tumours and 2–27% for ampullary tumours [1,2,7-10]. The large variation may partly be explained by underreporting of R1 resections due to non-standardized protocols for microscopic evaluation of the resection margins [9,11]. Furthermore, little is known about the relative importance of the different resection margins in R1 resections as determinants for survival [5,9,12]. The techniques employed for examination of the resected specimens clearly influence the reported rates of R0/R1 resections. Several groups have suggested guidelines for standardization of histopathologic assessment [13-19]. However, the retroperitoneal resection margin, which is most often involved in non-curative resections [5,13,20,21], is often not systematically evaluated in studies reporting histopathologic prognostic factors after pancreaticoduodenectomy [22-25].

The considerable variations in reported percentages of R1 resections for pancreatic and ampullary tumours may also be explained by difficulties in determining the cancer origin. Even after systematic histopathologic evaluation, the precise origin may be impossible to determine due to tumour destruction of normal periampullary anatomy [13,26-29]. There is also considerable normal variation of periampullary ductal structures, adding to the difficulties [26]. The common practice of reporting data on only a single periampullary subtype makes comparison of studies difficult due to the expected variations in inclusion and exclusion criteria for periampullary subtypes. For example, survival after resection of ductal pancreatic adenocarcinoma may be overestimated if ampullary cases are not adequately excluded [30]. Adjusted Cox regression analysis [31] including tumour origin as a covariate adjusts for some of the uncertainties regarding periampullary subtype classification, and also eliminates redundant or duplicate information resulting from associations between tumour origin and other covariates. Thus, we propose that survival analysis of all periampullary adenocarcinomas should include the tumour origin as a covariate rather than only presenting the results from separate subgroups.

Starting from 1998, we have employed a standardized protocol for evaluation of pancreaticoduodenectomy specimens, including serial perpendicular sectioning of the retroperitoneal resection margin and prospective evaluation of the cancer origin. The aim of this study was to investigate whether tumour involvement of the retroperitoneal margin is an independent prognostic factor for survival after resection of periampullary adenocarcinoma. Tumour origin was included as a covariate both in the overall adjusted analysis of all presumed curative (R1 and R0) periampullary resections and in a separate subgroup analysis of R0 resections.

## Methods

### Patient cohort

The study was approved by the National Committees for Research Ethics in Norway, project number S-05081, and was in compliance with the Helsinki Declaration. From 1998 to 2004, 161 consecutive patients underwent pancreaticoduodenectomy at the Department of Surgery, Rikshospitalet University Hospital, a third-level referral hospital. Of these, 114 patients (55 women and 59 men; median age 68 years; range 41–82) had primary adenocarcinoma with macroscopically free margins (R0 or R1 resections). Seventy six of the 114 included patients died before the end of the study, and the remaining 38 patients were followed up for a median of 4.8 years (range 1.6–8.4). None of the patients received preoperative chemo- or radiotherapy. During the study period, national guidelines did not recommend postoperative chemo- or radiotherapy. All patients underwent a standard Whipple's procedure including a distal gastrectomy. An effort was made to skeletonize the superior mesenteric and portal veins and the superior mesenteric artery in all cases, without performing extended lymphadenectomy. There were three cases with vascular resection (of which one tumour originated in the peripapillary duodenum and two were pancreatic). Intra-operative frozen sections from the bile duct and pancreatic neck resection margins were performed upon macroscopic suspicion of tumour involvement. Perioperative death (in-hospital death or death within 30 days of operation) was 3.5% (4/114). Cases with perioperative death were included in the survival analysis.

### Standardized protocol for examination of resection specimens

**Figure 1:**
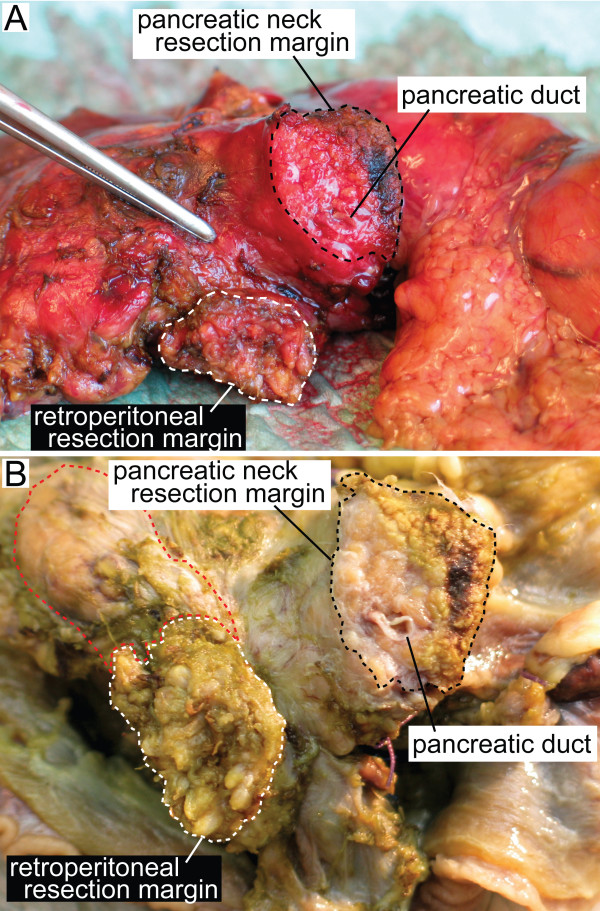
Close-up of the posterior aspect of a pancreaticoduodenectomy specimen from a patient with adenocarcinoma in the pancreatic head without tumour infiltration into the retroperitoneal margin, before (A) and after (B) fixation in formalin. The retroperitoneal resection margin was defined as the area of sharp dissection (white stapled area) in the peripancreatic fatty tissue behind the pancreatic head and lateral to the mesenteric vessels. In this case this area was relatively small. In cases with tumour infiltration or inflammation involving the posterior aspect of the pancreatic head, the size of this sharply dissected area may extend into the superior mesenteric vein groove (A, indicated by the forceps) or to a larger part of the posterior pancreatic surface (B, red stapled area). The black stapled area (A, B) indicates the pancreatic neck transection margin.

**Figure 2:**
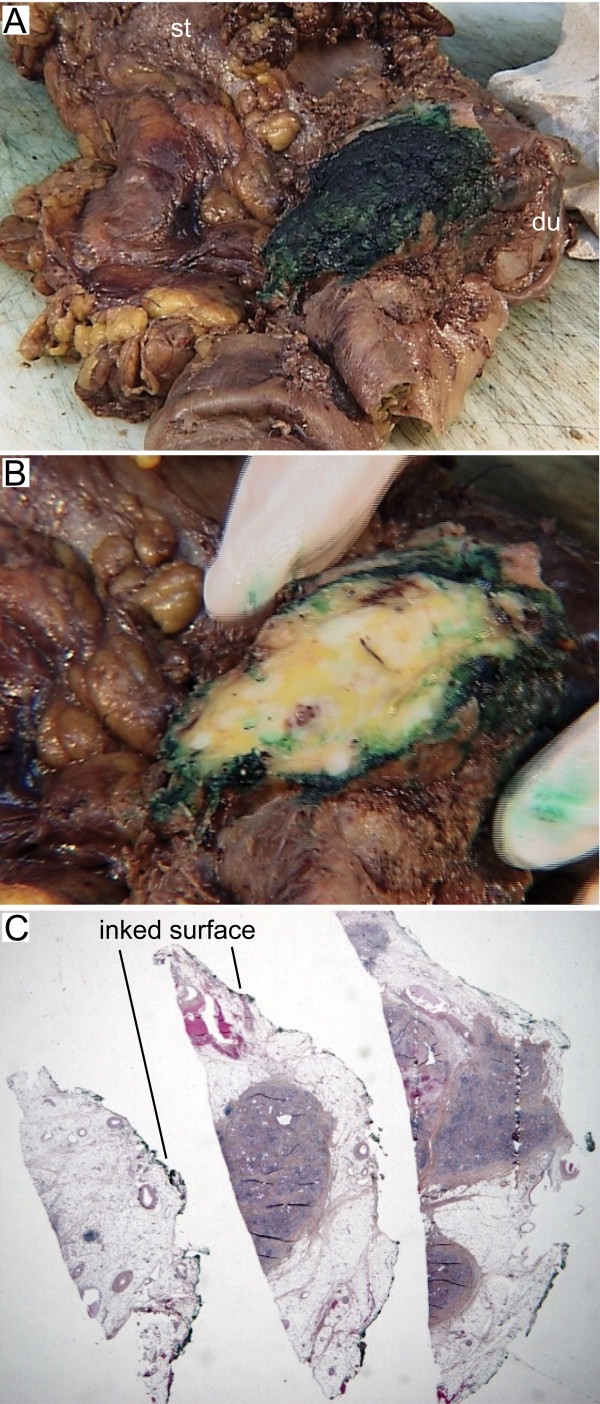
(A) Posterior view of a pancreaticoduodenectomy specimen with periampullary adenocarcinoma infiltrating the retroperitoneal resection margin. The specimen includes the pyloric part of the stomach (st), the duodenum (du), adipose tissue which is part of the greater omentum, and the head and uncinate process of pancreas. The retroperitoneal resection margin was identified and marked with ink. (B) A section parallel to this resection margin was made. (C) Perpendicular sections of the retroperitoneal resection margin demonstrate pancreatic parenchyma and connective tissue, including fat, vessels and nerves, with infiltration of tumour cells < 1 mm from the inked margin (visible on higher magnification), thus revealing a non-curative (R1) resection.

**Figure 3:**
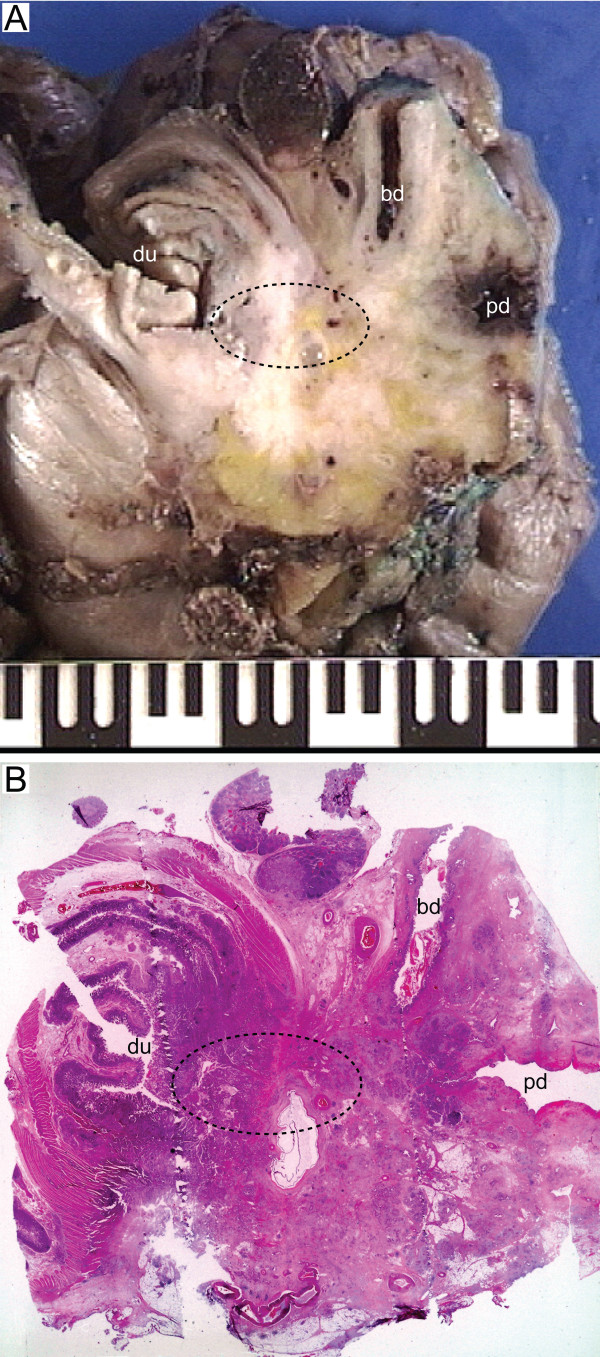
Whole-mount section parallel to the ampulla, distal bile duct and pancreatic duct, demonstrating periampullary tumour growth relative to ductal anatomy, for determination of the site of cancer origin. (A) White areas on the macroscopic photograph indicate possible tumour growth. (B) On microscopic examination, this tumour was found to originate from the peri-papillary duodenum (du), although it also involved the entire ampullary region (stapled area), and the distal portions of the distal bile duct (bd) and pancreatic duct (pd). In such cases, and in particular if epithelial dysplasia or in situ neoplasia also affects more than one periampullary sublocation, determination of the cancer origin may be difficult, and virtually impossible without systematic histopathologic examination.

In this study, we defined the retroperitoneal margin as the area of sharp dissection in the peripancreatic fatty tissue behind the pancreatic head and lateral to the mesenteric vessels (Figure 1). After fixation of the pancreaticoduodenectomy specimen in formalin, one block from the pancreatic neck and distal bile duct resection margins, respectively, was secured. These sections were taken parallel to the resection margins (shave sections). One block from the stomach and small bowel resection margins, respectively, was also secured. The retroperitoneal margin was identified and inked, and a section parallel to the resection margin (5–10 mm thick slice) was made, from which serial perpendicular sectioning into 5 mm thick slices was performed (Figure 2) [11,13]. The pancreatic duct and the distal bile duct, and their orifice(s) at the duodenal surface were identified, and probes were inserted in order to locate any obstruction within these ducts. A section parallel to the ductal structures, including duodenum, ampulla, distal bile duct and pancreatic parenchyma on a single slide, was made in order to demonstrate the tumour's relation to each of these potential sites of origin (Figure 3) [1]. Cross sections into the tumour were then made to evaluate tumour size and potential infiltration into adjacent structures. Lymph nodes were sampled from the duodenal knee and large and lesser curvatures of the stomach.

### Histopathologic evaluation of specimens

The following histopathologic factors were prospectively registered by routine examination: Tumour origin, maximum tumour diameter, degree of differentiation, perineural infiltration, vascular infiltration, dysplasia or other tumour associated pathologic changes, lymph node status, and resection margin status (pancreatic, bile duct, stomach, jejunal and retroperitoneal margins evaluated independently). All registrations were later reevaluated by an experienced pathologist. Finally, tumour origin was independently assessed by a second experienced pathologist. The cancer origin was determined by assessing tumour location relative to ductal anatomy and duodenal and pancreatic parenchyma, and by noting any associated epithelial dysplasia or in situ neoplasia. Upon disagreement, consensus was reached by discussion. All tumours were assigned to one of the four types using this approach. The final allocation of tumour origin corresponded with the initial prospective evaluation in 89 of 114 specimens (78%).

An R0 resection was defined as both macro- and microscopically free margins. An R1 resection was defined as tumour within 1 mm of a resection margin upon microscopic examination of haematoxylin and eosin stained sections. An R2 resection was defined as macroscopic residual tumour at the operative site, as described in the surgeon's operative report. Degree of differentiation was classified according to a two-score system as proposed by Lüttges et al. [32], distinguishing high-grade from low-grade carcinomas by presence or no presence, respectively, of areas with poorly differentiated tumour.

### Statistical analysis

Survival data were obtained from the National Registry of Norway. The Kaplan-Meier method was used to calculate curves for overall survival and to estimate median survival. Survival curves were compared using the log-rank test. Associations between categorical variables were examined using Chi-square test and Fisher's exact test. Mann-Whitney test and Kruskal-Wallis test were performed to compare tumour diameter (measured as a continuous variable) between groups of independent samples. The factors evaluated were: tumour origin, maximum tumour diameter, degree of differentiation, perineural infiltration, vascular infiltration, lymph node status, and resection margin status (pancreatic, bile duct, and retroperitoneal margins; no tumour infiltrated the stomach or jejunal resection margins).

Cox regression models were fitted in order to estimate unadjusted and adjusted survival after presumed curative (R0 and R1) resection, together with the hazard ratios with their 95% confidence intervals. For categorical variables, the group with the best prognosis in unadjusted analysis was set as reference. Hazards were proportional for all covariates, allowing inclusion of covariates in the adjusted analysis without need for stratification. All the examined histopathologic factors were significant in the unadjusted analysis and were thus included in the adjusted models. Two separate models were fitted for adjusted analysis of all histopathologic factors, considering the resection margins collectively (R1 versus R0) and individually (retroperitoneal margin free versus involved, and pancreatic margin free versus involved; omitting the stomach and duodenal resection margins, that had no cases with tumour involvement, and the distal bile duct margin, that had only two cases with tumour involvement). Factors were evaluated using forward stepwise variable selection, thus avoiding inclusion of variables with redundant prognostic information. In order to estimate the relative importance of the individual resection margins, the adjusted analysis was repeated after exclusion of the seven patients that had multiple margin tumour involvement. This analysis gave very similar results, with the same covariates in the final adjusted models as in the analysis including all 114 patients. These seven patients were thus not excluded from the analysis. A separate adjusted Cox regression subgroup analysis was performed for R0 resected patients in order to determine the factors that were independently associated with survival in curative resections. The proportional hazards assumption was evaluated by examination of log minus log plots (see Additional file 1: Verification of the proportional hazards assumption). Likelihood ratio test was computed to examine possible interactions between covariates (see Additional file 2: Evaluation of possible interaction between tumour origin and resection margin status).

All statistical analyses were performed with SPSS 15.0 for Windows software (SPSS Inc., Chicago, Illinois, USA). A two-sided p < 0.05 was considered statistically significant.

## Results

In this study of 114 macroscopically margin-free pancreaticoduodenectomies, 65% and 35% were R0 and R1 resections, respectively (Table 1). The retroperitoneal margin was involved by tumour infiltration in 80% of the R1 resections (32 of 40). Seven of the thirty-two tumours that infiltrated the retroperitoneal margin also infiltrated the pancreatic neck transection margin (of which two also infiltrated the distal bile duct margin). Resection margin involvement was significantly associated with each of the other prognostically poor histopathologic factors (regional lymph node involvement, p < 0.001; vessel infiltration, p = 0.001; perineural infiltration, p < 0.001; presence of areas with poor differentiation, p = 0.005; large tumour, p = 0.013). Resection margin involvement was most frequent when the tumour originated from the distal bile duct or pancreas (p = 0.009).

**Table 1:** Origin of tumour versus margin involvement and other histopathologic characteristics in 114 periampullary adenocarcinomas

	**Origin of tumour**				**Total** **(n = 114)**	**p-value**^a^
	Ampulla (n = 41)	Duodenum (n = 16)	Distal bile duct (n = 17)	Pancreas (n = 40)		
**Margin involvement**						0.009^b^
Any margin (R1 resections)	10	2	10	18	40	
Retroperitoneal	9	1	8	14	32	
Pancreatic neck	2	1	5	7	15	
Distal bile duct	0	0	0	2	2	
No margin (R0 resections)	31	14	7	22	74	
**Other histopathologic characteristics**						
Nodal status						0.005
N1	17	10	7	31	65	
N0	24	6	10	9	49	
Degree of differentiation						0.009
Poor	8	5	9	21	43	
High or moderate	33	11	8	19	71	
Vessel involvement						0.013
yes	9	4	9	21	43	
no	32	12	8	19	71	
Perineural infiltration						<0.001
yes	13	6	11	32	62	
no	28	10	6	8	52	
Tumour size						<0.001^c^
large (diameter ≥ 2.6 cm)	8	11	6	23	48	
small (diameter ≤ 2.5 cm)	33	5	11	17	66	

^a^Chi-square test, when not otherwise specified

^b^R1 vs R0 resection

^c^Measured as a continuous variable, Kruskal-Wallis test and Mann-Whitney test (pancreatic vs non-pancreatic)

### Unadjusted overall survival

**Figure 4:**
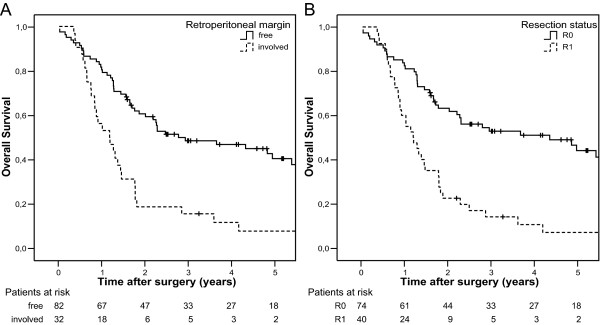
Overall survival after pancreaticoduodenectomy for periampullary adenocarcinoma (n = 114) with (A) free versus involved retroperitoneal resection margin (p < 0.001) and (B) R0 versus R1 resection (p < 0.001).

**Figure 5:**
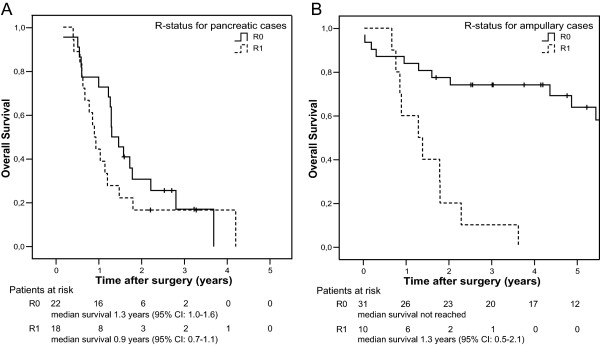
Overall survival for R0 versus R1 resection of tumours originating in (A) the pancreas (n = 40; p = 0.30) and (B) the ampulla (n = 41; p < 0.001).

In the unadjusted Cox regression analysis of 114 periampullary adenocarcinomas, tumour involvement of the resection margins predicted a poor prognosis compared to margin-free resections (Figure 4; see also Additional file 3: Unadjusted analysis of histopathologic prognostic factors), both when the resection margins were modelled collectively (R1 versus R0 resections, p < 0.001) and separately (retroperitoneal margin involved versus free, p < 0.001; pancreatic neck transection margin involved versus free, p = 0.003; bile duct resection margin involved versus free, p = 0.005). As expected, patients with cancer originating from the pancreas had the worst prognosis, with a median postoperative survival of 1.2 years (95% CI: 1.0–1.4), compared to 4.9 years (95% CI: 2.4–7.4) for ampullary tumours (p < 0.001). However, although the prognosis for R0 resected patients was significantly associated with tumour origin (p < 0.001), the prognosis after non-complete (R1) resections did not depend on tumour origin (p = 0.45). Comparing resections of pancreatic and ampullary tumours (Figure 5), resection status was found to be a more powerful predictor for survival for patients with ampullary tumour (p < 0.001, Figure 5B) than for patients with pancreatic tumour (p = 0.30, Figure 5A). Patients with ductal pancreatic adenocarcinoma had an estimated, statistically non-significant survival benefit of only five months for curative versus non-curative resection (p = 0.30, Figure 5A), while most of the patients with R0 resected ampullary tumours were still alive by the end of the study (19 of 31; median survival not reached, >5 years) and all patients with R1 resected ampullary tumours were dead by the end of the study (10 of 10) (p < 0.001, Figure 5B). The interaction between resection margin status and tumour origin for these two groups was statistically significant (p = 0.009).

### Adjusted analysis of presumed curative resections

In order to establish whether the retroperitoneal resection margin was an independent prognostic factor for clinically resectable periampullary adenocarcinomas (R0 and R1 resections), we performed adjusted Cox regression analysis including in a forward variable selection process all the variables that were significant in the unadjusted analysis. This resulted in the adjusted models (Table 2), in which tumour involvement of one or more resection margins (Table 2A), and the retroperitoneal margin in particular (Table 2B), independently predicted a poor prognosis after pancreaticoduodenectomy for periampullary adenocarcinoma (p = 0.010), adjusting for lymph node status and perineural infiltration (p < 0.01 for each, in both adjusted models).

**Table 2:** Adjusted Cox regression analysis of histopathologic prognostic factors (n = 114)

		HR	95% CI	p-value
**A. Model 1**				
Resection margin status	R1 (vs R0)	1.90	1.17–3.10	0.010
Lymph nodes	N1 (vs N0)	2.24	1.28–3.91	0.005
Perineural infiltration	yes (vs no)	2.22	1.31–3.75	0.003
**B. Model 2**				
Retroperitoneal margin	involved (vs free)	1.89	1.16–3.08	0.010
Lymph nodes	N1 (vs N0)	2.29	1.32–3.99	0.003
Perineural infiltration	yes (vs no)	2.32	1.38–3.92	0.002

HR, hazard ratio. HR > 1 indicates increased probability of death compared to the reference group

### Survival after microscopically margin-free resections

Finally, we performed a subgroup analysis of all patients that underwent a curative (R0) resection (n = 74). Pancreatic tumour origin was significantly associated with each of the other prognostically poor histopathologic factors (regional lymph node involvement, p = 0.008; vessel infiltration, p = 0.004; perineural infiltration, p < 0.001; presence of areas with poor differentiation, p = 0.001; large tumour, p < 0.001). Although these factors were significantly associated with survival in unadjusted analysis, adjusted analysis with stepwise forward variable selection resulted in a final model that included only tumour origin, which was thus the only independent predictor of survival after curative pancreaticoduodenectomy in the present study. The hazard ratio for R0 resected ductal pancreatic versus ampullary adenocarcinoma was 4.71 (95% CI: 2.13–10.4, p < 0.001). Median survival for patients with R0 resected pancreatic cancer was 1.3 years (95% CI: 1.0–1.6) while patients with R0 resected non-pancreatic cancer survived median more than 5 years (median survival not reached for ampullary and duodenal cases; p < 0.001).

## Discussion

Standardized protocols for evaluation of the resection margins should be mandatory in studies reporting prognostic data on periampullary adenocarcinomas [1,9,13,19,33]. The retroperitoneal margin should be evaluated by serial perpendicular sectioning [13]. Insufficient examination of the retroperitoneal margin might lead to underreporting of R1 resections[9,11]. Although most investigators report overall resection margin involvement to be an independent prognostic factor [2,3,7,8,34], some investigators have concluded otherwise [6,22]. Specific data on the retroperitoneal margin were not included in these reports. In the present study we have used a standardized systematic protocol for histopathologic assessment of resection margin involvement, with special attention to the retroperitoneal margin. Our main finding was that resection margin involvement, and retroperitoneal margin involvement in particular, independently predicts a poor prognosis in curative-intent (R0 and R1) resections for periampullary adenocarcinoma. In addition, we found that the anatomic tumour origin was the only independent prognostic factor in macro- and microscopic margin-free (R0) resections.

A problem when considering standardization of histopathologic reporting of pancreaticoduodenectomy specimens is that the definition of the retroperitoneal resection margin varies considerably. Some investigators define this margin simply as "the peripancreatic fat tissue behind the head of the pancreas [13,15]." Others include only the tissue directly adjacent to the proximal 3–4 cm of the superior mesenteric artery [16,19], sometimes with a clear distinction between the "retroperitoneal" and the "posterior pancreatic" resection margins [16]. The retroperitoneal margin is also often synonymously referred to as the "posterior," "mesenteric" or "uncinate" margin [14,17]. Some have advocated examination of the whole peripancreatic fatty tissue resection margin [20,21,35]. Verbeke et al. [9] recently evaluated a standardized protocol for examination of the circumferential resection margin, subdividing this margin into the anterior, posterior and superior mesenteric vein groove circumferential resection margins. In cases with inflammation and tumour invasion it may be difficult to distinguish between such distinct resection margins. Most important for evaluation of tumour margin infiltration is the area of sharp dissection, the extent of which varies depending on the degree of inflammation and tumour invasion. In our study, we thus widened the strictest definition of the retroperitoneal resection margin, but omitted separate analysis of each aspect of the circumferential peripancreatic margin in order to avoid extensive sampling.

The use of non-standardized protocols for histopathologic assessment may not only cause inconsistencies in the reporting of R0 versus R1 rates, but could also lead to differences with respect to classification of the anatomic site of tumour origin [1,13]. In the present study, tumour origin did not independently predict survival in presumed curative (R0 and R1) resections, although this factor was borderline significant when evaluated in a base model adjusting for all other histopathologic factors. There are probably two reasons for this. First, patients with pancreatic tumours (with the poorest prognosis in unadjusted analysis) frequently had resection margin involvement (45%). Thus, adjusting for resection margin status in the adjusted analysis renders tumour origin statistically non-significant. Second, in the unadjusted analysis, tumour origin was significantly associated with survival only in R0, not R1, resections. Consequently, in the adjusted analysis for R0 resected patients, tumour origin was the only histopathologic factor that independently predicted long-term survival after pancreaticoduodenectomy.

Interestingly, patients with ampullary adenocarcinoma had a considerable survival benefit of a retroperitoneal margin-free resection, while a free margin at this site was only non-significantly associated with survival for patients who had adenocarcinoma originating in the pancreas. Even when considering the resection margins collectively, we found only a non-significant tendency towards some five months benefit of having a margin-free resection in the pancreatic group. This is in line with previous reports, since the difference between median survival of patients with margin-free versus margin-involved resections from ductal pancreatic adenocarcinoma has typically been reported to be about half a year [5,8,9,36-38]. In a large, multicenter, prospective study of resected pancreatic cancer, Neoptolemos et al. [36] found that resection margin status was not an independent predictor of survival in ductal pancreatic adenocarcinoma. The retroperitoneal resection margin was however not systematically evaluated, and the R0 rate was exceptionally high (81%), possibly underestimating the rate of R1 resections [9]. In a study primarily comparing pancreaticoduodenectomy with or without vascular resection, Tseng et al. [37] reported that retroperitoneal margin involvement was not an independent prognostic factor in patients with pancreatic adenocarcinoma. However, stepwise variable selection was not performed, and the definition of the retroperitoneal margin was restricted to the area directly adjacent to the superior mesenteric artery. Evaluating individual resection margins in 160 resected pancreatic adenocarcinomas, Kuhlmann et al. [5] found that R0 resection independently predicted a favourable prognosis, but did not report the independent prognostic importance for survival of the retroperitoneal margin in particular. Thus, to establish whether or not involvement of the retroperitoneal resection margin independently predicts the prognosis also in ductal pancreatic adenocarcinoma, larger studies using standardized evaluation of both tumour origin and the individual resection margins should be performed.

## Conclusion

Systematic histopathologic evaluation confirms that resection margin involvement, and retroperitoneal margin involvement in particular, independently predicts a poor prognosis in curative-intent (R0 and R1) resections of periampullary adenocarcinoma. Involvement of the retroperitoneal margin is frequent in pancreatic, distal bile duct and ampullary tumours, and serial perpendicular sectioning of the retroperitoneal margin should thus be performed in all pancreatic head adenocarcinomas to avoid underestimation of R1 resections.

## Competing interests

The author(s) declare that they have no competing interests.

## Authors' contributions

AW participated in design of the study, registration and ethical approval, patient inclusion, review of clinical data, and histopathologic analysis. He also designed the database, performed the statistical analysis, and drafted the manuscript. ST participated in patient inclusion and had a major responsibility for histopathologic analysis. INF contributed with establishment of the protocol for systematic histopathologic assessment, participated in design of the study, patient inclusion, and histopathologic analysis. MC contributed substantially with choice of statistical methods and participated in statistical analysis. TJE participated in establishing systematic pathologic review of pancreaticoduodenectomy specimens, in design of the study, and in histopathologic analysis. ØM contributed substantially in the discussion of operative methods and performed many of the pancreaticoduodenectomies. OPC participated in design of the study, had a major responsibility for histopathologic analysis, and contributed substantially with critical review of the manuscript. IPG participated in design, registration and ethical approval of the research project, and in patient inclusion and registration of clinical data. He also performed many of the pancreaticoduodenectomies, contributed substantially in the discussion of statistical methods, and drafted the manuscript. All authors critically reviewed the manuscript and approved the final manuscript.

## Pre-publication history

The pre-publication history for this paper can be accessed here:



## Supplementary Material

Additional file 1 Verification of the proportional hazards assumption. Comparison of hazard ratios in the overall base model with hazard ratios obtained by stratification by each covariate, graphically illustrated by log minus log plots. Click here for file

Additional file 2 Evaluation of possible interaction between tumour origin and resection margin status. A possible interaction between tumour origin and resection status was evaluated by comparing likelihoods calculated for models with and without the interaction term, respectively. Click here for file

Additional file 3 Unadjusted analysis of histopathologic prognostic factors. Median survival and hazard ratios from unadjusted Cox survival analysis of histopathologic prognostic factors in all resections and in microscopic curative resections. Click here for file
