# Supplementary material for: Resectable adenocarcinomas in the pancreatic head: the retroperitoneal resection margin is an independent prognostic factor
Source: BMC Cancer. 2008 Jan 14;8:5. doi: 10.1186/1471-2407-8-5 (PMC2249586; doi:10.1186/1471-2407-8-5)

Verification of the proportional hazards assumption (n=114)

Graphical evaluation

(n=110; excluding perioperative mortality)

Covariates	HR	95.0% CI for HR
**Base model 1, without stratification**		
Origin		
- duodenal vs. ampullary	0.57	0.21 - 1.52
- distal bile duct vs. ampullary	0.51	0.23 - 1.17
- pancreatic vs. ampullary	1.19	0.62 - 2.31
Resection margin status	1.82	1.09 - 3.06
Lymph node status	1.55	0.84 - 2.86
Poor differentiation	1.82	1.06 - 3.12
Vessel involvement	1.72	1.02 - 2.92
Perineural infiltration	1.66	0.92 - 3.02
Tumour diameter (in cm)	1.23	0.96 - 1.58

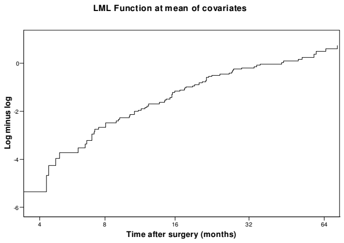

**Base model 1, stratified by tumour origin**		
Resection margin status	1.74	1.03 - 2.93
Lymph node status	1.72	0.90 - 3.27
Poor differentiation	1.83	1.06 - 3.15
Vessel involvement	1.65	0.96 - 2.84
Perineural infiltration	1.59	0.86 - 2.93
Tumour diameter (in cm)	1.21	0.94 - 1.57

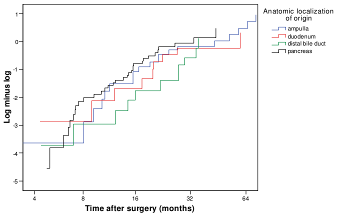

**Base model 1, stratified by resection status**		
Origin		
- duodenal vs. ampullary	0.57	0.21 - 1.52
- distal bile duct vs. ampullary	0.52	0.22 - 1.18
- pancreatic vs. ampullary	1.14	0.58 - 2.24
Lymph node status	1.58	0.85 - 2.94
Poor differentiation	1.91	1.11 - 3.28
Vessel involvement	1.66	0.97 - 2.84
Perineural infiltration	1.62	0.90 - 2.95
Tumour diameter (in cm)	1.22	0.95 - 1.56

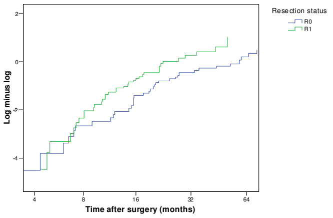

**Base model 1, stratified by lymph node status**		
Origin		
- duodenal vs. ampullary	0.51	0.18 - 1.42
- distal bile duct vs. ampullary	0.50	0.22 - 1.14
- pancreatic vs. ampullary	1.21	0.62 - 2.35
Resection margin status	1.85	1.10 - 3.12
Poor differentiation	1.90	1.11 - 3.27
Vessel involvement	1.77	1.04 - 3.00
Perineural infiltration	1.66	0.91 - 3.01
Tumour diameter (in cm)	1.19	0.91 - 1.54

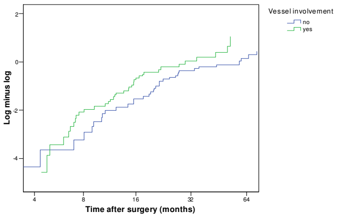

**Base model 1, stratified by vessel infiltration**		
Origin		
- duodenal vs. ampullary	0.56	0.21 - 1.47
- distal bile duct vs. ampullary	0.56	0.24 - 1.28
- pancreatic vs. ampullary	1.17	0.60 - 2.28
Resection margin status	1.74	1.03 - 2.94
Lymph node status	1.53	0.83 - 2.82
Poor differentiation	1.75	1.02 - 3.02
Perineural infiltration	1.71	0.94 - 3.13
Tumour diameter (in cm)	1.23	0.96 - 1.58

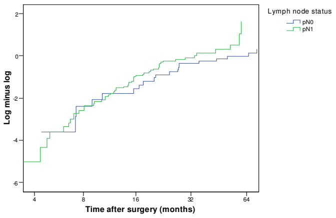

**Base model 1, stratified by perineural infiltration**		
Origin		
- duodenal vs. ampullary	0.57	0.21 - 1.54
- distal bile duct vs. ampullary	0.50	0.22 - 1.14
- pancreatic vs. ampullary	1.18	0.61 - 2.30
Resection margin status	1.84	1.10 - 3.09
Lymph node status	1.50	0.82 - 2.75
Poor differentiation	1.95	1.13 - 3.37
Vessel involvement	1.75	1.03 - 2.98
Tumour diameter (in cm)	1.24	0.96 - 1.60

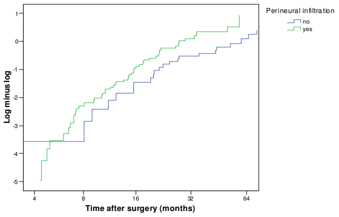

**Base model 1, stratified by presence of poor differentiation**		
Origin		
- duodenal vs. ampullary	0.62	0.23 - 1.70
- distal bile duct vs. ampullary	0.54	0.24 - 1.24
- pancreatic vs. ampullary	1.16	0.59 - 2.29
Resection margin status	1.86	1.10 - 3.13
Lymph node status	1.50	0.81 - 2.77
Vessel involvement	1.67	0.98 - 2.86
Perineural infiltration	1.73	0.95 - 3.16
Tumour diameter (in cm)	1.21	0.94 - 1.55

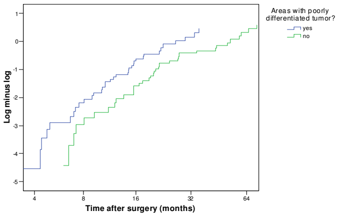

Supplement: Additional file 1 — Verification of the proportional hazards assumption. Comparison of hazard ratios in the overall base model with hazard ratios obtained by stratification by each covariate, graphically illustrated by log minus log plots. [file 1471-2407-8-5-S1.pdf]
