# Supplementary material for: Resectable adenocarcinomas in the pancreatic head: the retroperitoneal resection margin is an independent prognostic factor
Source: BMC Cancer. 2008 Jan 14;8:5. doi: 10.1186/1471-2407-8-5 (PMC2249586; doi:10.1186/1471-2407-8-5)
Supplement: Additional file 2 — Evaluation of possible interaction between tumour origin and resection margin status. A possible interaction between tumour origin and resection status was evaluated by comparing likelihoods calculated for models with and without the interaction term, respectively. [file 1471-2407-8-5-S2.pdf]

Evaluation of possible interaction between tumour origin and resection margin status

A. Analysis including all four tumour origins (ampulla, duodenum, distal bile duct, pancreas) and resection margin status (free vs involved) (n=114)

	df	p-value	HR	95.0% CI for HR Lower	Upper
Covariates (assuming no interaction)					
R-status	1	0.000	2.61	1.59	4.27
Origin	3	0.002			
Origin(duodenum vs ampulla)	1	0.699	1.17	0.54	2.54
Origin(distal bile duct vs ampulla)	1	0.652	0.84	0.40	1.78
Origin(pancreas vs ampulla)	1	0.002	2.50	1.41	4.44
-2 Log Likelihood of model coefficients					
607.31	4				
Covariates (assuming interaction)					
R-status	1	0.000	6.03	2.51	14.48
Origin	3	0.000			
Origin(duodenum vs ampulla)	1	0.387	1.51	0.59	3.84
Origin(distal bile duct vs ampulla)	1	0.972	0.98	0.28	3.47
Origin(pancreas vs ampulla)	1	0.000	4.72	2.19	10.20
Origin*R-status	3	0.103			
Origin(duodenum vs ampulla)*R-status	1	0.493	0.54	0.09	3.19
Origin(distal bile duct vs ampulla)*R-status	1	0.437	0.53	0.11	2.61
Origin(pancreas vs ampulla)*R-status	1	0.014	0.25	0.08	0.75
-2 Log Likelihood of model coefficients					
601.24	7				
Change in Chi-square					
607.31 - 601.24 = 6.07	3	0.10<p<0.20*			

B. Analysis including the two largest groups of tumour origin (ampulla, pancreas) and resection margin status (free vs involved) (n=81)

	df	p-value	HR	95.0% CI for HR Lower	Upper
R-status	1	0.000	7.21	2.74	18.97
Origin	1	0.000	5.81	2.43	13.89
Origin*R-status	1	0.009†	0.20	0.06	0.67

* non-significant

† significant
