# Supplementary material for: Resectable adenocarcinomas in the pancreatic head: the retroperitoneal resection margin is an independent prognostic factor
Source: BMC Cancer. 2008 Jan 14;8:5. doi: 10.1186/1471-2407-8-5 (PMC2249586; doi:10.1186/1471-2407-8-5)
Supplement: Additional file 3 — Unadjusted analysis of histopathologic prognostic factors. Median survival and hazard ratios from unadjusted Cox survival analysis of histopathologic prognostic factors in all resections and in microscopic curative resections. [file 1471-2407-8-5-S3.pdf]

Unadjusted analysis of histopathologic prognostic factors

		No of patients	Median survival (years)	HR	95% CI for HR	p-value
**A. R0 and R1 resections (n=114)**						
R-status	R0 (ref)	74	4.4			
	R1	40	1.2	2.82	1.78–4.49	< 0.001
Retroperitoneal margin	free (ref)	82	2.8			
	involved	32	1.2	2.61	1.63–4.18	< 0.001
Pancreatic neck margin	free (ref)	99	2.2			
	involved	15	1.2	2.45	1.35–4.43	0.003
Distal bile duct margin	free (ref)	112	1.9			
	involved	2	0.6	8.27	1.88–36.4	0.005
Origin	ampulla (ref)	41	4.9			< 0.001
	duodenum	16	2.3	1.09	0.50–2.38	0.822
	distal bile duct	17	2.5	1.20	0.58–2.49	0.615
	pancreas	40	1.2	3.15	1.79–5.53	< 0.001
Lymph nodes	N0 (ref)	49	NR			
	N1	65	1.3	3.30	1.97–5.53	< 0.001
Poor differentiation	no (ref)	71	3.7			
	yes	43	1.2	2.59	1.64–4.11	< 0.001
Vessel involvement	no (ref)	71	3.7			
	yes	43	1.3	2.80	1.76–4.47	< 0.001
Perineural infiltration	no (ref)	52	5.4			
	yes	62	1.3	3.17	1.93–5.19	< 0.001
Tumour size (continuous)	diameter (cm)	114	1.8	1.27	1.08–1.48	0.003
**B. R0 resections (n=74)**						
Origin	ampulla (ref)	31	NR			< 0.001
	duodenum	14	5.0	1.51	0.59–3.84	0.389
	distal bile duct	7	NR	1.00	0.28–3.55	0.998
	pancreas	22	1.3	4.71	2.13–10.4	< 0.001
Lymph nodes	N0 (ref)	41	NR			
	N1	33	1.7	2.89	1.51–5.55	0.001
Poor differentiation	no (ref)	53	5.4			
	yes	21	1.3	2.94	1.54–5.62	0.001
Vessel involvement	no (ref)	54	5.4			
	yes	20	1.3	2.43	1.24–4.75	0.009
Perineural infiltration	no (ref)	43	6.0			
	yes	31	1.7	2.38	1.27–4.48	0.007
Tumour size (continuous)	diameter (cm)	74	4.3	1.23	1.00–1.50	0.045

HR, hazard ratio. HR > 1 indicates increased probability of death compared to the reference group

ref, reference for categorical variables

NR, not reached
